# Ecology of Pollen Storage in Honey Bees: Sugar Tolerant Yeast and the Aerobic Social Microbiota

**DOI:** 10.3390/insects14030265

**Published:** 2023-03-08

**Authors:** Kirk E. Anderson, Brendon M. Mott

**Affiliations:** Carl Hayden Bee Research Center, USDA Agricultural Research Service, Tucson, AZ 85719, USA

**Keywords:** fungus, yeast, hive environment, beebread, microbiota, pollen consumption, evolution, xerophilic, acidophilic

## Abstract

**Simple Summary:**

Historically, the storage of collected pollen by honey bees was thought to rely on microbes to enhance pollen nutrition. However, this hypothesis has found little empirical support. More recent experiments that quantified pollen storage time, microbial load relative to pollen mass, and variation in the microbiota clearly indicate that honey bees do not rely on microbial enzymes to alter the nutritional quality of collected pollen. Here, we quantified abiotic factors that suppress microbial growth in stored pollen and determined microbial abundance relative to pollen mass using both culturing and molecular assays. We found that microbial growth is quickly suppressed by added honey- and host-supplied enzymes, but that sugar tolerant yeasts subsist longer than bacteria in stored pollen. This work contributes to our understanding of host–microbial interactions in the honey bee and highlights the aerobic social microbiota, a symbiotic and omnipresent collection of native bacteria and yeasts that dominate the social resource space of the honey bee colony and hive.

**Abstract:**

Honey bee colonies are resource rich and densely populated, generating a constant battle to control microbial growth. Honey is relatively sterile in comparison with beebread: a food storage medium comprising pollen mixed with honey and worker head-gland secretions. Within colonies, the microbes that dominate aerobic niches are abundant throughout social resource space including stored pollen, honey, royal jelly, and the anterior gut segments and mouthparts of both queens and workers. Here, we identify and discuss the microbial load in stored pollen associated with non-*Nosema* fungi (primarily yeast) and bacteria. We also measured abiotic changes associated with pollen storage and used culturing and qPCR of both fungi and bacteria to investigate changes in stored pollen microbiology by both storage time and season. Over the first week of pollen storage, pH and water availability decreased significantly. Following an initial drop in microbial abundance at day one, both yeasts and bacteria multiply rapidly during day two. Both types of microbes then decline at 3–7 days, but the highly osmotolerant yeasts persist longer than the bacteria. Based on measures of absolute abundance, bacteria and yeast are controlled by similar factors during pollen storage. This work contributes to our understanding of host–microbial interactions in the honey bee gut and colony and the effect of pollen storage on microbial growth, nutrition, and bee health.

## 1. Introduction

Although the vast majority of bee species live solitary lives, social species such as the honey bee dominate the pollination landscape. The life history transition of corbiculate bees from solitary/annual to social/perennial involved highly selective fine tuning of microbial control and social immunity [1,2,3,4]. Two of four primary features of eusociality, overlapping generations and cooperative brood care, are tied to complex behavioral traits that evolved under continuous pressure from both opportunistic and infectious disease [5]. However, in both solitary and social bees, microbes co-opted from the floral environment have been demonstrated to inhibit aerobic opportunists and provide protection for developing larvae and stored pollen [6,7,8,9,10,11].

Considered ancestral behaviors shared with social species, solitary female bees collect pollen, mix it with mechanically dehydrated nectar, form the mixture into a ball, and top that ball with a single fertilized egg, then protect the investment with plant material, hollows, or select soil types [12]. As the solitary bee larvae grows and develops, it competes with microbes to consume the pollen and nectar provisions. This life-history strategy generated an intimate fitness association of bees with the bioactive properties of the floral and nesting environment [13,14,15]. Trained and/or transmitted by the floral environment, various aerobic microbes compete to consume the pollen ball including opportunistic pathogens (ubiquitous molds) and potentially beneficial microbes that suppress mold growth such as *Lactobacillus*, Acetobacteraceae, and sugar tolerant yeasts [16,17]. Disease caused by mold is common, and solitary bee species have evolved great variation in physiology and behavior to combat mold growth or limit its spread to neighboring larvae [12], including the addition of plant resins and salivary secretions. Generally, the microbial function of these nectar-rich pollen balls resembles that of silage production, wherein acid generated by fermentative metabolism of simple sugars inhibits opportunistic mold growth at the interface with oxygen.

As a pinnacle of social evolution, the honey bee wields far greater control of its microbial environment [2]. Complex social organization has resulted in a well-guarded hive nursery filled with ultra-concentrated food sources and precise host control of their preparation and maintenance. Stored pollen, also called “beebread”, begins as balls of collected corbicular pollen carried on the legs of foragers. Already mixed with honey, these pollen balls are firmly packed into wax cells by worker bees [8,18]. After packing, the beebread appears dull and powdery, but after a day or two, it is capped with a shiny layer of honey, rendering the contents anaerobic. In contrast to solitary bee larvae, honey bee larvae do not consume pollen directly, but are fed a nutrient rich jelly secreted from adult head glands, a group-living strategy that intimately connects the microbiology of the colony [19,20]. Both honey and jelly are highly antimicrobial according to different mechanisms [21,22] and serve to mitigate the microbial challenges associated with perennial group living including extended food storage and exposed larval development [19,23].

In this contribution, we investigate the nature of bacteria and yeasts in beebread, the colony niche harboring the greatest microbial and enzymatic diversity [8,24]. Although many studies have speculated that pollen in beebread is predigested, providing essential nutrients [24,25], a highly comprehensive and conclusive study revealed no predigestion and, moreover, a conspicuous lack of conditions required for predigestion, such as pollen storage time [8]. Honey bee workers preferentially and quickly consume freshly collected pollen [8,18,26], with most collected pollen being consumed within 1 week and the remainder within 2–3 weeks [26]. In choice tests, honey bees preferred one-day-old pollen [18]. Consistent with earlier findings, beebread consumption by workers is far too rapid for any type of systematic (co-evolved) predigestion by microbes.

Nevertheless, the honey bee hosts a highly predictable and niche-specific set of microbes that consistently populate the anaerobic hindgut [27] and aerobic social resource space of the colony and hive, including all nutrition storage or processing niches [3,10,25]. While the hindgut bacteria are facultative anaerobes, the microbiota that dominates the social environment can proliferate with exposure to oxygen, honey, propolis, and royal jelly. Prevalent members include the bacteria *Apilactobacillus kunkeei, Bombella apis*, and *Fructobacillus fructosus*, all acting to preserve and protect the hosts’ investment in fitness by populating developing larvae, stored food, and associated host tissues such as mouthparts and midguts of queens and workers [23,28]. These microbial symbionts are exclusive to the genus *Apis* (honey bees) but all share a recent common ancestor that populates the floral environment [6,7,29,30]. As a testament to their co-evolved nature, all three of these bacterial species increase significantly throughout social resource space when the colony is exposed to propolis [3].

As the reservoir for both aerobic and anaerobic microbiota, beebread is 50% honey by weight [15]. Much of the preservative properties of beebread are provided by the abiotic properties of honey and the addition of host enzymes. When added to nectar, glucose oxidase secreted by the workers results in a chemical reaction that produces hydrogen peroxide and gluconic acid (glucono-lactone), increasing the free hydrogen ion concentration and lowering the pH of honey below pH 4 [31,32]. This chemical reaction requires H_2_O and O_2_ and is associated with a mechanical dehydration of nectar, a worker behavior known as bubbling: the repeated transfer of nectar from the social stomach to the mouthparts, mixing in host enzymes, and exposing the viscous liquid to the atmosphere. This mechanical dehydration and chemical reaction happens quickly, before the forager even returns to the hive [33]. The most common bacteria in beebread and honey have evolved to tolerate this osmotic and oxidative stress, and grow very quickly, producing acid via fermentation of glucose and fructose. Within a week, the beebread environment becomes overly toxic and microbes either die or enter a state of enzymatic stasis [34]. Consistent with this model, a couple studies using next-generation sequencing have revealed a wealth of microbial diversity in beebread, but microscopy and molecular work demonstrates that microbes are sparse and fungal hyphae are absent in 1-week-old beebread [8]. However, the addition of a small amount of water to beebread causes a rapid bloom of various microorganisms, primarily yeasts [35].

## 2. Materials and Methods

### 2.1. Monitoring Pollen Deposition and Age

Colonies maintained at apiaries in Tucson, AZ, USA were provided additional top boxes with empty drawn comb and wax foundations to allow beebread deposition. Based on experience, we designed our sampling effort to procure sufficient pollen cells of known age and restrict most bees from consuming the known-age pollen before it could be sampled. Thirteen colonies were selected for monitoring of stored pollen that was to be collected and packed over the next 24 h. A frame from each colony was selected that was part of, or adjacent to, the brood area, had open brood, already contained some stored pollen, but had sufficient open space for new pollen deposition. The pattern of stored pollen present when the experiment began was scored by overlaying a transparent acrylic sheet and circling cells of pollen present. A cell was considered filled when the bottom of the cell was completely covered with pollen. On subsequent days of monitoring, a cell was considered empty if the bottom of a marked cell was visible. Colonies were scored and sampled over the course of a week. Having identified the newly deposited pollen, we used push-in cages made from hardware cloth (2 × 3 inches in size) to sequester the newly deposited pollen from further deposition. Ten or so young nurse bees were allowed to remain with the cells under the cages during this period.

### 2.2. Abiotic Factors Affecting Pollen Preservation

To provide environmental context for culturing and microbial enumeration, we determined the pH and water content associated with naturally collected pollen as a function of storage time. As detailed above, we tracked stored pollen “beebread” by known storage age, measuring the pH with an electrode designed to quantify semi-solid samples (pH spear from Eutech Industries) and water content by desiccation and subtraction. We, first, weighed the beebread sample, and then desiccated the sample in a drying oven (Precision Scientific), determining the difference in weight attributable to water. We, then, assessed the relationship of beebread age with both pH and water content using regression analysis of log transformed data, performed in either Sigma Plot or SAS [36].

### 2.3. Culturing Beebread in Spring and Summer Dearth

We examined the change in beebread microbiotas: differentiating yeast, molds, and bacteria in beebread by number and type. To estimate the population density of each major type, we combined high-resolution light microscopy (ZEISS, Dublin, CA, USA) with standard plate counts, examining variously aged beebread. Beebread was monitored and sampled according to Anderson et al. [8], prior to packing (corbicular pollen removed from the legs of foragers), and following packing at two and six days of age in both August of 2014 and April of 2015. In August and April, we monitored eight and thirteen colonies, respectively, to quantify microbe type and absolute abundance in beebread aged 0–7 days. To determine beebread age, the pattern of beebread on a chosen frame was recorded by overlaying a transparent acrylic sheet and circling cells with pollen present, then using a colored marker as in Anderson et al. [8]. We, then, tracked frames daily for newly filled cells, and beebread age was defined using a variety of colors.

To quantify microbial colony-forming units (CFUs), we plated replicate samples on both plate count agar (PCA) and Sabaroud dextrose agar (SDA), media with neutral and acidic pH that support a broad spectrum of fungal growth [11]. In addition to fungus, these media support the growth of three of the most prominent co-evolved hive bacteria typically found in beebread, *Apilactobacillus kunkeei*, *Bombella apis* (previously *Parasaccharibacter apium*), and *Fructobacillus fructosus,* the major contributors to fermentation of hive food stores, beebread, and honey [11]. We quantified microbial growth via plate counts in spring 2015 using PCA and SDA with and without two added antibiotics (chloramphenecol (12.5 μL/mL) and ceftazidime (5 μL/mL)).

Beebread samples of various known age were cored with straws and suspended in 600 μL of physiological saline (0.9% NaCl, 0.1% Tween 80, 0.1% Peptone). Triplicate plates containing antibiotics or not were produced from this initial suspension. We used the remaining 300 μL to produce triplicate serial dilutions. As the source of beebread cultures, corbicular pollen pellets (one from each forager) were suspended in 400 μL physiological saline, vortexed for 5 min on medium speed, and plated without dilution. After three days of growth, we scored and counted the plates. Following log transformation of the data, we used t-tests or ANOVA to compare CFU abundance across time periods performed in either Sigma Plot or SAS [36].

### 2.4. Microscopic Identification

Four replicate plates were examined separately for each of the time periods. Following culturing, microbial colonies were picked from the petri dish and visually designated as bacteria or fungi according to their morphology under light microscopy at 1000×. The plate was first bisected four times to create eight equal-sized areas, and 10 microbial colonies were picked and examined from each of these eight areas (*n* = 80 per plate). CFUs were confirmed as bacterial, mycelial, or yeast based on shape and size. Yeasts were of discernible shape, showing budding characteristics and observable nuclei under the greatest magnification (1000×). CFUs were scored as mold if mycelial structure was present under low magnification and/or if mycelia present had characteristics such as obvious branching or septa. Bacterial CFUs were discernible only at the greatest magnification, were coccoid or rod shaped, had no observable nuclei, no budding characteristics, and were non-branching. Actinobacteria were assumed from the following collection of characteristics: mycelial-like structures observable at the greatest magnification, no nuclei or sporulation characteristics, and cells roughly the same width as a typical rod-shaped bacteria. When plates contained >300 CFUs, the proportion of colonies in the same section exhibiting the same morphology were classified as the same organism based on the law of large numbers. If 30 of 300 CFUs chosen at random are all type A, this reflects the strong probability that the remaining CFUs are type A.

### 2.5. Sampling Long-Term Pollen Storage

In a second related experiment, we quantified the ratio of fungi to bacteria in beebread following eight weeks of storage over the winter months. As source material, we sampled eight healthy 8–10 frame colonies maintained just south of Tucson, AZ, USA near Santa Rita, AZ, USA. We used a sterile straw to remove six beebread cores from each of the eight colonies just prior to December 7, repeating this sampling eight weeks later (February 8). Beebread cores were sampled to represent a variety of frame locations within the hive relative to the expected center of the overwintering cluster (high/low/inside/outside). There were no available sources of pollen during this time, and the beebread present within the hive was caged with hardware cloth to prohibit worker consumption but allow ambient temperature and humidity produced by colony respiration.

#### 2.5.1. Isolating Microbial DNA

DNA was isolated from pooled beebread cores following methods to concentrate and separate microbial DNA from stored pollen grains. We pooled 6–10 core samples prior to DNA extraction, then normalized to 0.25 g of stored pollen for the DNA extraction procedure. We isolated DNA according to Anderson et al. 2014 [8] with the following changes: Following the addition of lysis buffer to the samples, they were bead beaten with a Mini-Beadbeater-16 (BioSpec #607, Bartlesville, OK, USA) for 30 s, then moved to an ice bath for 30 s. This cycle was repeated two additional times for a total of 90 s of mechanical disruption by bead-beating. We then extracted DNA using the GeneJet Genomic DNA Purification Kit (Thermo Scientific #K0722, Waltham, MA, USA) following the protocol for Gram-positive bacteria.

#### 2.5.2. Estimating Microbial Load of Long-Term Pollen Storage

We estimated the size of both bacterial and fungal communities in beebread using degenerate bacterial primers and qPCR accompanied by a dilution series of known plasmid standards. To quantify bacteria, we first extracted total genomic DNA from non-transformed DH5α™ cells (*E. coli*). The 16s gene template was amplified using forward primer 27F (5′-AGAGTTTGATCCCTCAG-3′) and reverse primer 1522R (5′- AAGGAGGTGATCCAGCCGCA-3′). For fungal quantification, total genomic DNA was extracted from *S. cerevisiae* cells. The 18s gene template was amplified using forward primer PanFungal_18S_F (5′-GGRAAACTCACCAGGTCCAG-3′) and reverse primer PanFungal_18S_R (5′-GSWCTATCCCCAKCACGA-3′). This primer set does not amplify the ubiquitous microsporidian *Nosema*. We created plasmid vectors using Invitrogen’s pCR^TM^2.1 TOPO^TM^ cloning vectors per the manufacture’s specifications. Ligated vectors were then transformed into DH5α™ cells per the manufacture’s specifications. Transformed colonies were selected and grown overnight in broth. Cells were then pelleted out and the plasmid DNA was purified using the Thermo Scientific GeneJET Plasmid Miniprep Kit (#K0503). The mass of a single plasmid molecule was calculated per the formula provided by Applied Biosystems. An Implen nanophotometer P300 was used to assess DNA concentration of the purified plasmid solution and subsequent 10-fold serial dilutions were made. The dilutions were then used as the standards for the qPCR quantification. See Liu et al. (2012a) and Liu et al. (2012b) for additional information on Bactquant and Fungiquant molecular assays [37,38]. Following log transformation, we performed t-tests comparing time periods and regression analysis examining the ratio of fungi to bacteria using either Sigma Plot or SAS [36].

## 3. Results

### 3.1. Abiotic Factors of Early Beebread Storage

**Figure 1:**
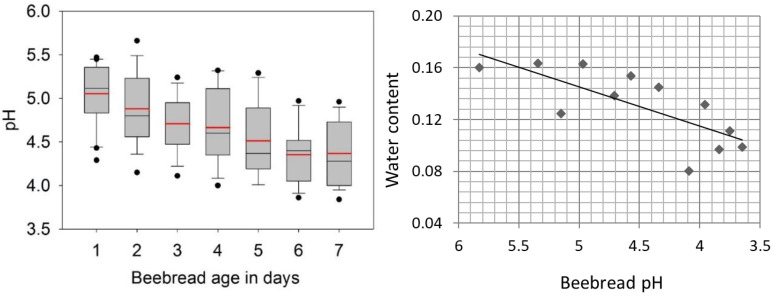
Abiotic factors associated with pollen storage that mitigate microbial growth. The figure on the left represents the variation in pH from stored pollen “beebread” from seven hives tracked by beebread age. The red line is the mean, black the median, boxes are at 25% and 75%, whiskers at 5% and 95%, and dots are outliers. Beebread pH decreased significantly over the seven-day period following pollen collection by foragers. The panel on the right is a subset of beebread samples that represent variation in pH and its relationship to water availability. The proportion of water available in beebread decreased significantly as a function of storage time and decreasing pH.

We measured pH and water content of stored pollen or “beebread” over the first seven days of storage. Beebread pH decreased significantly over the seven-day period following pollen collection by foragers (t_36_ = 5.9, *p* < 0.00001). The proportion of water available in the beebread also decreased significantly over the assessed period as a function of storage time and decreasing pH (Figure 1). We found a significant negative association of pH with water availability, explaining half of the variation in the model (Adjusted R^2^ = 0.51, F_2,10_ = 12.6, *p* < 0.005) and indicating that the progressive water loss is significantly associated with an increase in hydrogen ion concentration.

### 3.2. Microbial Growth in Beebread

**Figure 2:**
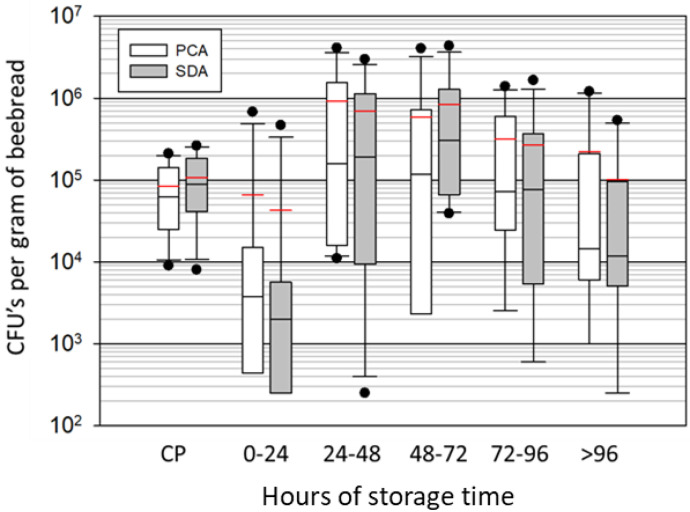
Total microbial growth in August (primarily yeast and bacteria) from fresh corbicular pollen (CP) and beebread sampled daily for 5 days at 24 h increments. Identical samples were plated in triplicate on standard plate count agar (PCA) and Sabouraud dextrose agar (SDA). Each box plot displays the sampled variation from eight colonies. The red line is the mean, black the median, boxes are at 25% and 75%, whiskers at 5% and 95%, and dots are outliers. Abundance differs significantly by time (F_5,66_ = 2.98, *p* < 0.01).

Our culturing results show an initial steep decline in abundance from corbicular pollen to 1-day-old beebread (Figure 2). From one to two days, both bacteria and fungi grew steeply and significantly to their highest levels, and then leveled off, with both fungal and bacterial numbers declining steadily from 3 to 6 days. Based on an ANOVA of log transformed values, their abundance differs significantly by time (F_5,66_ = 2.98, *p* < 0.01). Microbial load decreased significantly when comparing the peak of growth (2 days) to the values obtained after six days of storage (t_22_ = 2.1, *p* < 0.05).

### 3.3. Distinguishing Microbial Type

**Figure 3:**
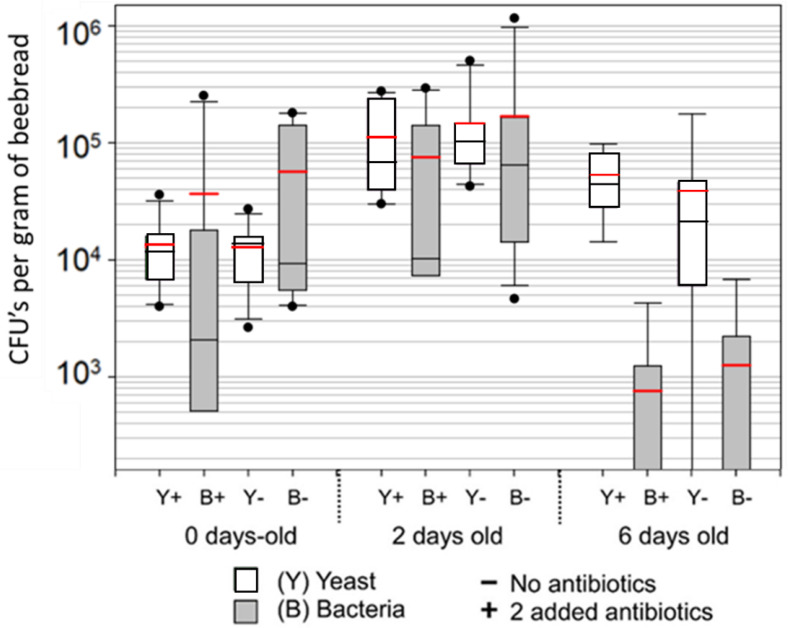
Culture dependent results from colony build-up during spring bloom. Using a dilution series, and Sabouraud dextrose agar, we cultured fungal and bacterial growth from fresh corbicular pollen (0 days old) and pollen stored for 2 or 6 days. Box plots display variation in microbial growth across 13 colonies sampled over 6 days. The red line is the mean, black the median, boxes are at 25% and 75%, whiskers at 5% and 95%, and the dots are outliers. Samples were cultured with (+) and without (−) the addition of antibiotics chloramphenecol and ceftazidime. Following growth, microorganisms (CFUs) were exhaustively identified as bacteria (grey) or yeast (white) using light microscopy.

Despite the use of media tailored to fungal versus bacterial organisms, we observed similar growth of both bacteria and fungus on both plate count agar and Sabouraud dextrose agar, including bacterial growth on plates spiked with antibiotics (Figure 3). When the growth medium contained no antibiotics, bacterial colonies outnumbered fungal colonies in corbicular pollen and fresh beebread but not in six-day-old beebread. Again, the abundance of both fungi and bacteria increased by an order of magnitude from corbicular pollen to 2 days of age, and then decreased significantly from 2 to 6 days of age.

**Figure 4:**
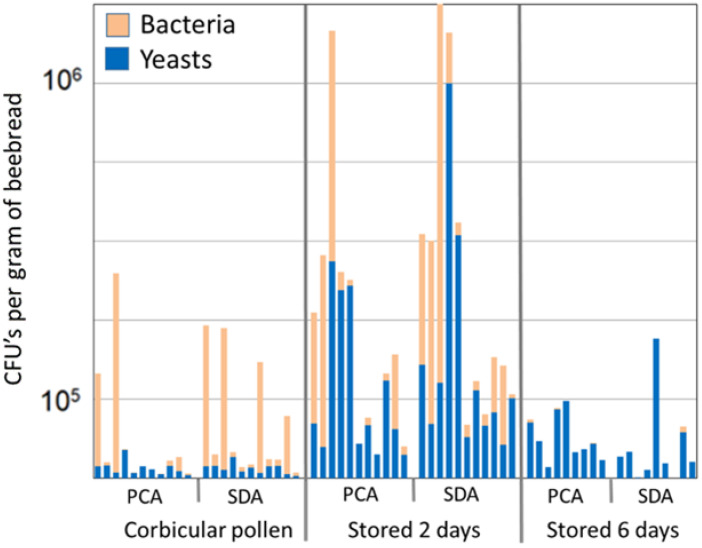
The relative ratio of yeasts and bacteria accounting for 99% of identified CFUs. Identical samples were plated on standard plate count agar (PCA) and Sabouraud dextrose agar (SDA) and grown aerobically. Isolates were identified by high-resolution light microscopy from 11 colonies during spring colony growth.

Initially, bacterial blooms outnumbered fungal colonies, and then, from two to six days of age, yeasts were cultured in significantly greater quantities than were bacteria (Figure 4). Yeast colonies accounted for the vast majority (>99%) of total fungal counts overall.

### 3.4. Long-Term Pollen Storage: FungiQuant and BactQuant

**Figure 5:**
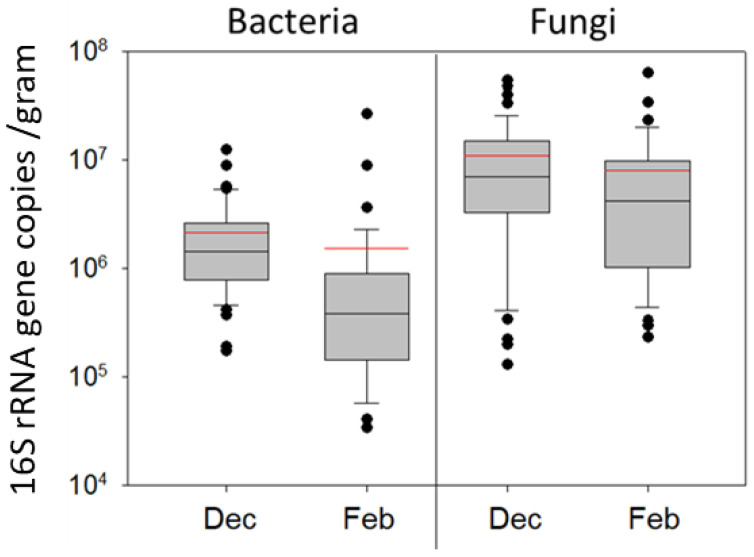
Abundance of bacteria and fungi in beebread calculated with qPCR of the rRNA gene and plasmid standards. Results display 80 beebread cells sampled from eight colonies on the seventh of December (Dec) and again on the eighth of February (Feb). Box plots display the variation in microbial abundance. The red line is the mean, black the median, boxes are at 25% and 75%, whiskers at 5% and 95%, and dots are outliers. Comparing log-transformed 16S rRNA gene copy number estimates, bacterial load decreased significantly overwinter (t_80_ = 4.9, *p* < 0.00001), but fungal load remained similar (t_80_ = 1.5, *p* = 0.14).

**Figure 6:**
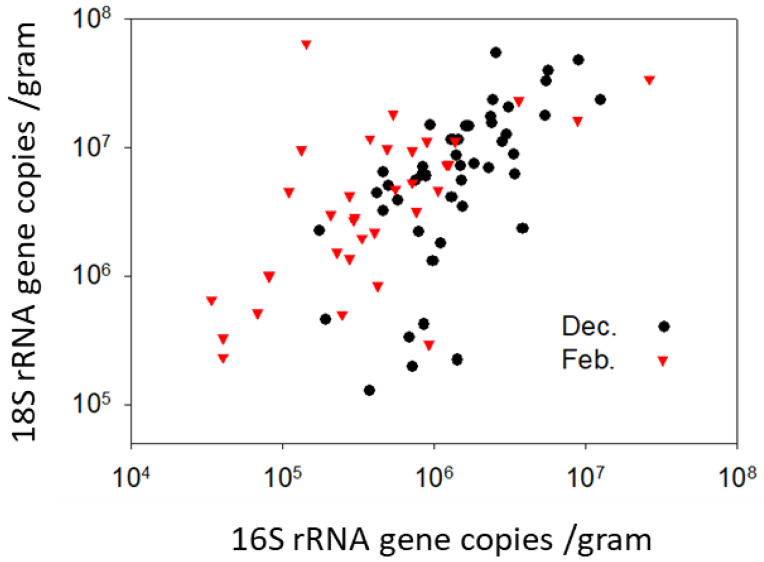
Data from Figure 5 illustrating the strong relationship between fungi and bacteria in stored pollen. The abundance of fungi (18S rDNA) and bacteria (16S rDNA) were significantly and positively correlated based on log-transformed estimates of abundance (R^2^ = 0.25, F_2,72_ = 24.2, *p* < 0.0001).

Fungi were detected at greater copy numbers than bacteria in both December and February (Figure 5), but following the transformation of copy number to cell number (CFUs), our qPCR values were roughly consistent with our culturing results. Budding yeasts are known to have 100–150 copies of rRNA genes per cell, while bacteria have far fewer (4.2 on average). Based on regression analysis of log-transformed cell count estimates (Figure 6), bacterial and fungal (yeast) loads in beebread were highly correlated in both December (R^2^ = 0.41, F = 31.2, *p* < 0.0001) and at the height of the winter forage dearth just prior to spring bloom in February (R^2^= 0.39, F = 20.7, *p* < 0.0001).

## 4. Discussion

Social resource space in a honey bee colony includes nutrient processing: stored food and larval feeding. The honey bee has evolved a variety of specialized mechanisms to cope with pathogen challenge throughout social resource space [1,39]. Our results confirm that beebread storage is associated with the rapid formation of an extreme acidophilic and xerophilic microenvironment evolved to preserve nutrition by limiting microbial growth [8,33]. Both pH and water availability drop steadily and rapidly for up to seven days post pollen packing. Quickly after collection, beebread becomes highly acidic at the interface with oxygen, partly the result of GOX producing hydrogen peroxide and gluconic acid [40] but also through the production of organic acids by lactic acid bacteria and sugar-tolerant yeasts. Over the first seven days of storage, water availability in beebread decreases significantly below the level required by most microbial life [41]. Microbes found with prevalence and abundance throughout the nutrition-processing network include the aerobic social microbiota, pathosphere bacteria at low prevalence and abundance, and core gut bacteria of workers and queens, all of which survive the beebread medium with variable success to be transmitted to new adult generations [42]. Based on past results and lab experience sequencing isolates from stored pollen, these bacteria are mostly *Bombella apis, Fructobacillus fructosus*, and *Apilactobacillus kunkeei* with lesser amounts of Enterobacteraceae and core gut bacteria *Lactobacillus* firm5, *Frischella*, *Gilliamella*, and *Bifidobacterium* [11,43]. Thus, beebread represents a microbial “seedbank” comprising native gut bacteria and yeasts, protective social symbionts including those that populate the queen, and various opportunistic microbes that survive over the long term via sporulation or other desiccation-resistant mechanisms.

Consistent with previous results characterizing beebread [34,44], we found that yeasts are the dominant microbial cell type and subsist longer than lactic acid bacteria during beebread storage, a testament to their co-evolved nature. This indicates that the explosion of CFUs in the first few days of pollen storage as recorded in previous work [8] is due in large part to the growth of yeasts, but not mold, in the pollen. This process of preservation may continue past seven days as our trendline suggests. We identified very few filamentous fungi in early beebread with our limited culture time, but results indicate that a variety of filamentous and mycotoxin-producing fungi can be found in beebread with some regularity [45].

We found strong correspondence between our two methods of quantification, with culture-dependent results returning similar cell counts as molecular results. The vast majority of fungi cultured from beebread were yeasts, but we found a greater ratio of fungi/bacteria using the DNA-based approach. Critically, 99% of fungal CFUs that grew on SDA and PCA media were identified as yeast when examined under a microscope, suggesting that the majority of rRNA genes identified using the FungiQuant assay were also yeast. The sugar-tolerant yeasts that occur in beebread are highly specialized commensal fungi [44,46,47], and some use polyols to counteract the osmotic pressure generated by high sugar concentrations [48]. Consistent with other systems, culture-dependent methods often fail to confirm various fungi implicated by PCR of the 18S rRNA gene [49]. All recorded estimates of microbial abundance in beebread are consistent with very low microbial biomass relative to available surface area [8]. Other estimates are similar for bacterial abundance [50], but fungal (yeast) abundance throughout social resource space remains to be verified.

The yeasts enumerated in this study [28,44,51] belong to the native and aerobic social microbiome [52], ubiquitous throughout social resource space including mouthparts, crops, midguts, and larvae and food stores [3,28,42,53,54]. Our findings agree with the conclusions of Tauber et al. [46,55] that yeasts (perhaps many different species) are constitutive functional members of the honey bee microbiota recycled by food storage and processing. Based on positive correlations with the core ileum bacteria, we hypothesize a system wherein symbiotic commensal yeasts are niche specialists similar to the bacterial gut symbionts [56]. Consistent with our findings, the efficient and fast growing aerobic microbiota of beebread inhibits the growth of other less favorable fungi and bacteria, many of which are vectored from floral or water sources. Following an initial drop in microbial abundance after corbicular pollen was packed into the wax cell, we saw a rapid bloom of honey-tolerant native bacteria and yeasts, primarily aerobes. This aerobic interface represents a hostile and highly selective xerophilic and acidophilic microenvironment wherein the availability of moisture, atmospheric oxygen, and bee-supplied enzymes results in a layer of oxidative activity that kills or inhibits the growth of most microbes. This effect is analogous to the “respiratory burst”, a cellular-level immune response of mammals and invertebrates that mitigates microbial growth using a targeted release of reactive oxygen species [57].

## Data Availability

Details are available by email request to the corresponding author.
